# Synthesis of innovative and sustainable gelatin*@*graphene oxide-crosslinked-zirconium silicate*@*gelatin nanobiosorbent for effective biosorption of basic fuchsin dye

**DOI:** 10.1038/s41598-023-31584-x

**Published:** 2023-04-01

**Authors:** Mohamed E. Mahmoud, Gehan M. Nabil, Sarah M. Elsayed, Amal R. Rashad

**Affiliations:** 1 Chemistry Department, Faculty of Sciences, Alexandria University, P.O. Box 426, Ibrahimia, 21321 Alexandria Egypt; 2 Chemistry Department, College of Arts and Science, Prince Sattam Bin Abdelaziz University, Wadi Eldawasser, Riyadh, Saudi Arabia; 3 Department of Modeling and Simulation, Advanced Technology and New Materials Research Institute (ATNMRI), City of Scientific Research and Technological Applications (SRTA-City), New Borg El-Arab City, 21934 Alexandria Egypt

**Keywords:** Environmental sciences, Chemistry, Materials science, Nanoscience and technology

## Abstract

Most dye stuffs and coloring materials are mainly categorized as hazardous pollutants in water effluents due to their nature as non-biodegradable, highly toxic and extremely carcinogenic. For this reason, rapid and efficient eradication of waste dyes from wastewaters before discharging into water streams must be accomplished by an acceptable approach as adsorption technique. Therefore, the present study is aimed and devoted to synthesize a novel nanobiosorbent from three different constituents, gelatin (Gel) as a sustainable natural product, graphene oxide (GO) as an example of highly stable carbonaceous material and zirconium silicate (ZrSiO_4_) as an example of combined metal oxides for the formation of Gel@GO-F-ZrSiO_4_@Gel by using formaldehyde (F) as a cross-linkage reagent. Several characterization techniques as FT-IR were employed to identify the incorporated surface reactive Functionalities in Gel@GO-F-ZrSiO_4_@Gel as –OH, =NH, –NH_2_, –COOH and C=O, etc. The morphology for particle shape and size of Gel@GO-F-ZrSiO_4_@Gel were confirmed from the SEM and TEM analyses providing 15.75- 32.79 nm. The surface area was determined by the BET and found to correspond to 219.46 m^2^ g^-1^. Biosorptive removal of basic fuchsin (BF) pollutant as an example of a widely applicable dye in various activities was monitored and optimized under the influence of pH (2–10), reaction time (1–30 min), initial BF pollutant concentration (5–100 mg L^−1^), nanobiosorbent dosage (5–60 mg), temperature (30–60 °C) and interfering ions. The maximum biosorptive removal values of BF dye were established as 96.0 and 95.2% using 5 and 10 mg L^−1^, respectively at the recommended pH 7 condition. The Thermodynamic parameters demonstrated that the BF dye adsorption onto Gel@GO-F-ZrSiO_4_@Gel was taken place via spontaneous and endothermic reaction. Chemisorption is the predominant adsorption mechanism by forming multilayers upon nonhomogeneous surface in accordance with Freundlich model hypothesis. The applicability of the optimized Gel@GO-F-ZrSiO_4_@Gel in biosorptive removal of BF pollutant from real water sample was successfully accomplished by the batch technique. Thus, this study clearly shows that Gel@GO-F-ZrSiO_4_@Gel exhibited significant influences on remediation of industrial effluents containing BF pollutant with superior efficiency.

## Introduction

Environmental pollution is well known and documented as a subject of major serious subject with high global concern^1^. Therefore, water pollution is classified as a topic of these with attracted significant number of research studies owing to its great effect and impact on humans, animals and plants lives^2^. Water pollution is generally caused by dumbing wastewater containing a great number pollutants from different origins including solid and liquid objects^3^, chemical and biological pollutants^4^, toxic heavy metals and radioactive isotopes^5^ organic and inorganic materials^6^, as well as other types of contamination^7^. Contamination of water with organic pollutants may be produced from humic substances^8^, phenolic derivatives^9^, petroleum wastes^10^, surfactants^11^, pesticides^12^, fertilizers^13^, pharmaceuticals^14^ and dyes as well as other organic contaminants^15^. Due to the high annual world production of dye stuffs (about 1,000,000 tons) for the sake of application in a number of important industrial fields as cosmetic, tanneries, textile, food and medicinal sectors, a great amount of contaminated wastewater dyes are generally dumped without prior treatment in water stream^16^. It has been reported that the textile sector plays a pertinent role in annual discharge of more than 7.5 tons to water system^17^. The majority of coloring materials and dyes are mainly known as hazardous pollutants due to their nature in view of non-biodegradability and high toxicity and carcinogenicity due to the presence of benzidine, phenylene and azo-moieties^18^. Henceforth, suitable methodologies for acceptable and rapid eradication of waste dyes from wastewaters before discharging into water streams and resources must be searched and ruled^19^.

Several approaches have been implemented and reported for wastewater treatment and dyes effluents with the high appreciation of adsorption technology in dye removal processes^20^. Therefore, enormous studies have been investigated and documented in adsorptive dye removal using a variety of adsorbents as activated carbon^21^, biochars^22^, metal–organic frameworks^23^, metal derivatives^24^, nanomaterials and nanocomposites^25^ and natural materials^26^. The specifications of adsorbent as specific surface area, surface functionality, porosity and recyclability contribute a major role in the application and removal process of dyes from wastewaters^27^. Gelatin is among the natural materials as a produced natural biopolymer from the cattle and pig collagen in a mixture of peptides and proteins and therefore, it has good functionalities and therefore, it has been employed in the assembly of some efficient adsorbents for removal of some toxic pollutants^28–31^. On the other hand, graphene and its derivatives have been applied in management processes of water pollution via adsorption technique^32^. Graphene oxide (GO) is generally produced by oxidation of graphene and therefore, it is characterized by different related functionalities of the incorporated oxygen in the forms of oxygenated as carboxylic, carbonyl and hydroxyl groups to enable GO for greater potency to undergo surface functionalization^33,34^. Therefore, GO with its existence in the nano-sized dimension has been categorized as a good candidate for preparation of various adsorbent via combination with other organic and inorganic derivatives^35–38^. Metal silicates as ZrSiO_4_ represent examples of highly stable materials with low solubility in different solvents have implemented in a well-known number of applications^39^. Therefore, ZrSiO_4_ was investigated and implemented in various nanocomposites as potential adsorbents in treatment of water from different pollutants^40–42^. Based on the outlined characteristics of gelatin, GO and ZrSiO_4_ with respect to the incorporated functional groups in these three materials, the aim of this study is devoted to design and assemble a novel nanobiosorbent via efficient combination reaction and covalent bonding. Three synthetic steps were followed in this study to assemble the aimed nanobiosorbent. The first step is devoted to prepare gelatin-loaded-graphene oxide (Gel@GO). The second step is directed to fabricate gelatin-loaded-zirconium silicate (Gel@ZrSiO_4_). The third step is related to simply crosslinking Gel@GO with Gel@ZrSiO_4_ by using formaldehyde for the formation of the target Gel@GO-F-ZrSiO_4_@Gel nanobiosorbent. Confirmation via instrumental characterization of Gel@GO-F-ZrSiO_4_@Gel was accomplished by a number of techniques as FT-IR, SEM, TEM, TGA, XRD and surface area. Basic fuchsin (BF) pollutant is as an example of a widely applicable cationic dye in various important activities including tracking proteins in acidic pHs, distinguish E.coli from K.aerogenes bacteria, detection and staining procedures as a nuclear stain, elastic tissue stain, and mucin stain. Therefore, the fourth step in the current study is mainly aimed and focused to explore the potential capability and affinity of the assembled Gel@GO-F-ZrSiO_4_@Gel nanobiosorbent for removal of BF pollutant from water via batch biosorption technique.

## Experimental

### Materials and chemicals

All of the utilized chemicals in the provided work are of analytical grade and employed without further purification as listed Table 1S (Supplementary materials).

### Instrumentations

The employed characterization techniques in this study with their specifications are tabulated in Table 2S (Supplementary materials).

### Synthesis of Gel@GO, Gel@ZrSiO_4_ and Gel@GO-F-ZrSiO_4_@Gel

**Figure 1:**
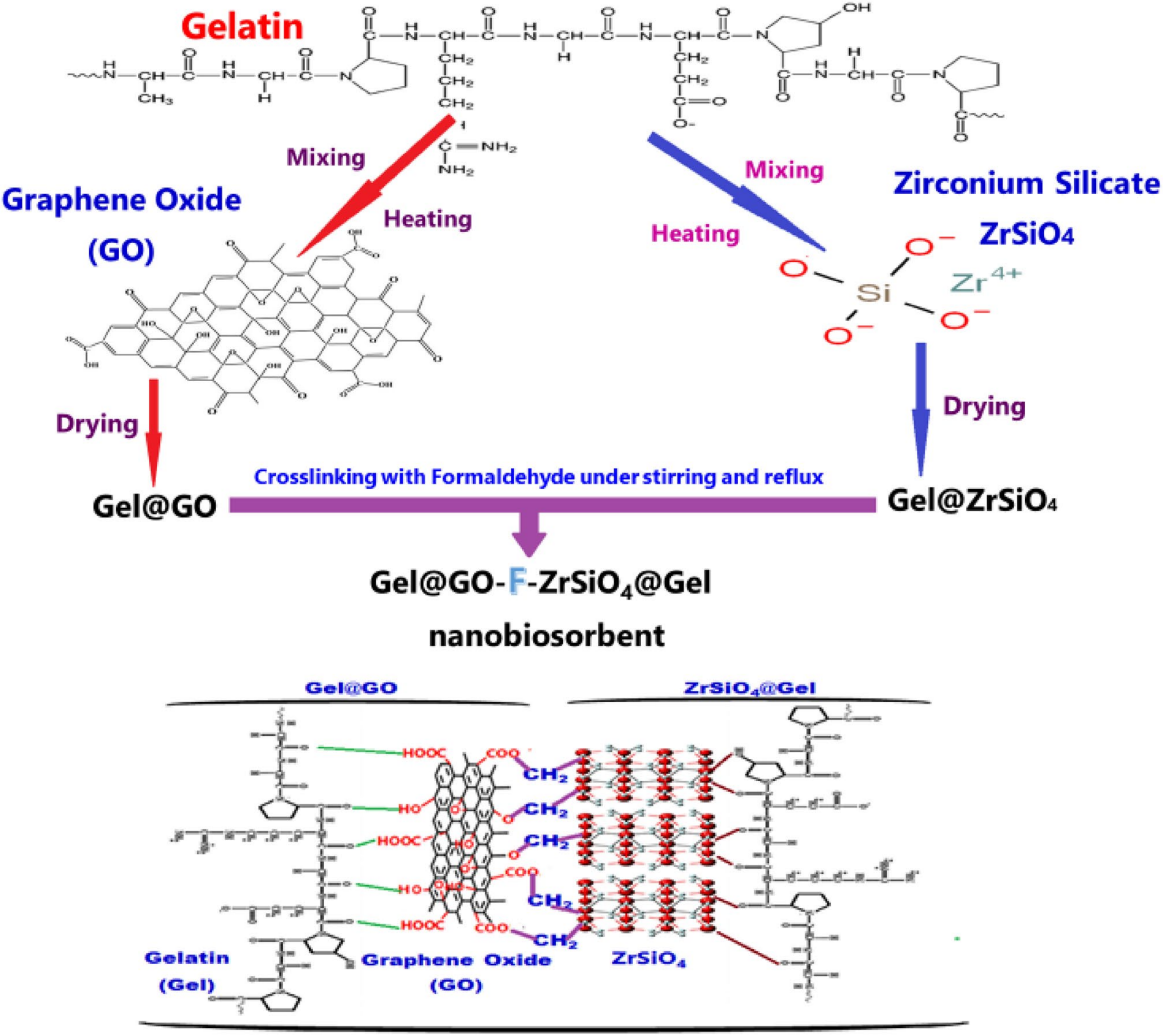
Synthesis of Gel@GO-F-ZrSiO_4_@Gel.

The suggested and proposed Gel@GO-F-ZrSiO_4_@Gel nanobiosorbent was assembled by following three successive steps. The chemical and 3D-structures of various constituents in Gel@GO-F-ZrSiO_4_@Gel nanobiosorbent are provided in Table 1. Firstly, gelatin-loaded-graphene oxide (Gel@GO) was prepared by initial fabrication of GO via the modified Hummers method using graphite powder, sodium nitrite, hydrogen peroxide (30%), sulfuric acid (70%), and potassium permanganate (99%) as previously described solubility in different solvents have implemented in a well-known number of applications^34^. A sample of gelatin (2.0 g) was then added to GO (2.0 g) in 20 mL distilled water (DW). The mixture was heated with stirring at 60 °C to a paste formation and 20 mL DW was then added. This mixture was subjected to addition of DW and heating as described and this process was repeated four times to produce the aimed Gel@GO material and dried at 50° C in an oven. Secondly, gelatin-loaded-zirconium silicate (Gel@ZrSiO_4_) was prepared according to the following procedure. Gelatin (2.0 g) was mixed ZrSiO_4_ (2.0 g) and 20 mL DW. This mixture was heated with stirring at 60 °C to a paste formation. Additional 20 mL DW was then added and heating and this process was repeated four times to produce the aimed Gel@ZrSiO_4_ material and dried in an oven at 50 °C. Thirdly, Gel@GO-F-ZrSiO_4_@Gel nanobiosorbent was assembled by the chemical reaction and cross-linkage of Gel@GO (2.0 g) with Gel@ZrSiO_4_ (2.0 g) using 50 mL of formaldehyde under steady stirring and reflux at 110 °C for 6 h. The resultant grey precipitate of Gel@GO-F-ZrSiO_4_@Gel nanobiosorbent was filtered before being dried in an oven at 50 °C. Figure 1 represents a schematic diagram for the assembly of the various nanobiosorbent to form the final product (Gel@GO-F-ZrSiO_4_@Gel)_._

**Table 1:** Chemical and 3D-structures of various constituents in Gel@GO-F-ZrSiO_4_@Gel.

Name (Abbreviation)	Chemical structure	3-D structure
Gelatin (Gel)		
Graphene oxide (GO)		
Zirconium silicate (ZrSiO_4_)

### Batch removal experiments

Stock solution of basic fuchsin pollutant (BF dye) was individually prepared by dissolving a certain amount in 1.0 L DW to produce 100 mg L^−1^ from this stock solution, the desired concentrations of BF dye (5 and 10 mg L^−1^) were prepared by further dilutions. A UV spectroscopy equipment was being used to figure out the absorbance value of BF dye at λ_max_ = 546 nm. The measurements were done at room temperature (25 °C), with the reactions being carried out by an automated shaker. The removal percent (R%) of adsorbed BF onto Gel@GO-F-ZrSiO_4_@Gel was determined from Eq. (1)**.**

1 $${\text{R}}\% = \left( {{\text{C}}_{{\text{o}}} - {\text{C}}_{{\text{e}}} /{\text{C}}_{{\text{o}}} } \right) \, \times { 1}00$$

wherein, C_o_ and C_e_ are the BF dye concentration (mg L^−1^) at time zero and time t, respectively.

The effects of Gel@GO-F-ZrSiO_4_@Gel dosage, pH, contact duration, initial dye concentration, interfering salts and temperature on BF removal were investigated and optimized in this study. Therefore, a series of batch adsorption experiments, via automated shaker were performed in an attempt to evaluate the adsorption performance of Gel@GO-F-ZrSiO_4_@Gel nanobiosorbent as well as finding out the most favorable conditions that achieve the maximum sorption potential. (i) The contribution of pH on the removal process of BF dye was examined by mixing 15 mg of Gel@GO-F-ZrSiO_4_@Gel and automatically shaking of 20 mL solutions of dye (5 and 10 mg L^−1^) in the range from pH 2–10, utilizing (1.0, 0.1 and 0.01 mol L^−1^) NaOH or HCl. After completion of the reaction, the samples were centrifuged for 20 min and the absorbance values of the mother liquors were determined with a UV–Vis at λ_max_ = 546 nm. (ii) The influence of Gel@GO-F-ZrSiO_4_@Gel dosage was investigated in the entire range (5—60 mg) by mixing 20 mL solutions of BF dye (5 and 10 mg L^−1^) and different masses of nanobiosorbent and shaking by an automatic shaker for 30 min and centrifuged for 20 min. The absorbance values of the mother liquor were determined by a UV–Vis at λ_max_ = 546 nm. (iii) The effect of contact reaction time and kinetic study were performed by shaking on an automatic shaker the reaction mixture of 20 mL dye solutions (5 and 10 mg L^−1^) with 15 mg of Gel@GO-F-ZrSiO_4_@Gel for durations ranging from 1 to 30 min. Following centrifugation, the absorbance values of leftover dye quantities were determined by a UV-Vis at λ_max_ = 546 nm. (iv**)** The adsorption isotherm modeling and impact of various BF dye concentrations on behavior of Gel@GO-F-ZrSiO_4_@Gel were accomplished by mixing 20 mL of 5.0 – 100 mg L^−1^ solutions with of 15 mg dosage Gel@GO-F-ZrSiO_4_@Gel. After shaking for 30 min, the mixture was centrifuged, and the values of absorbance were characterized from a UV-Vis analysis at λ_max_ = 546 nm. (v) The thermodynamic parameters and thermal effect under various reaction temperatures (30–60 °C) were investigated by reacting 25 mg dosage of Gel@GO-F-ZrSiO_4_@Gel with 20 mL of the BF dye (5 and 10 mg L^−1^) and automatically shaken for 30 min. Finally, the concentration of unadsorbed BF dye after centrifugation were characterized from a UV-Vis analysis at λ_max_ = 546 nm. (vi) The effect of interfering cation on adsorptive removal of BF dye onto Gel@GO-F-ZrSiO_4_@Gel was performed by reacting 20 mL of dye concentrations (5 and 10 mg L^−1^) with 100 mg of competing ions as Ca(II), Mg(II), NH_4_(I), K(I), and Na(I) with 15 mg of Gel@GO-F-ZrSiO_4_@Gel. After shaking for 30 min, the mixtures were centrifuged, and the absorbance values of were identified from a UV spectroscopic determination at λ_max_ = 546 nm.

### Application of Gel@GO-F-ZrSiO_4_@Gel nanobiosorbent on BF removal from water resources

The removal efficiency and capability of Gel@GO-F-ZrSiO_4_@Gel nanobiosorbent of BF-dye from real water resources was also accomplished in this work. Samples of wastewater, sea water and drinking water were collected and employed to prepare samples spiked with 5.0 and 10.0 mg L^−1^of BF. 20 mL of each sample was mixed and treated with a 15 mg of Gel@GO-F-ZrSiO_4_@Gel nanobiosorbent. Finally, after shaking for 30 min, the investigated samples were centrifuged, and the absorbance values were identified from a UV spectroscopic determination at λ_max_ = 546 nm.

## Results and discussions

### Characterization of the assembled Gel@GO-F-ZrSiO_4_@Gel nanobiosorbent

**Figure 2:**
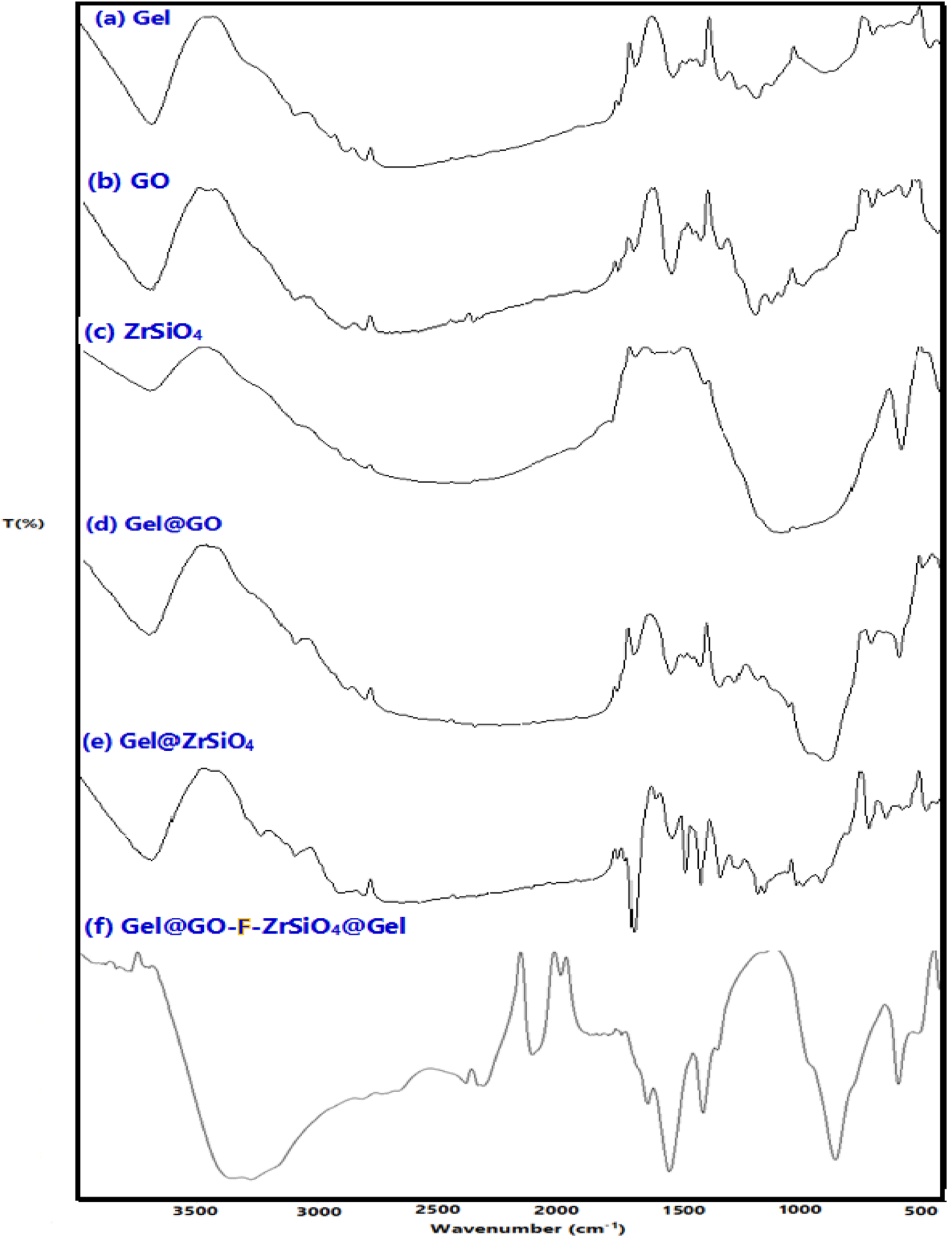
FT-IR of Gel@GO-F-ZrSiO_4_@Gel and various constituents.

To characterize and confirm the key functional groups in the as-prepared Gel@GO-F-ZrSiO_4_@Gel nanobiosorbent and types of chemical bonding, the FT-IR analysis was acquired. The assembled Gel@GO-F-ZrSiO_4_@Gel nanobiosorbent was prepared from the combination of three different constituents, i.e. gelatin, graphene oxide and ZrSiO_4_ and therefore, the FT-IR spectrum of this nanobiosorbent (Fig. 2) is expected to show several related peaks to GO at 462.38, 1049.36, 1234.76, 1366.52, 1581.34, 1713.18 and 3330.06, cm^−1^. The peak at 3330.06 cm^−1^ is caused by the physically adsorbed water molecules providing bending and stretching O–H groups in GO. In addition, the C–O vibration band exhibited two weak peaks at 1713.18 and 1581.34 cm^−1^, indicating the carboxylic acid or carbonyl groups in GO. The bending of aromatic C–H bonds in GO were assigned by the peaks at 1366.52 and 462.38 cm^−1^. The epoxy and carbonyl groups in GO were assigned by the peaks at 1234.76 and 1049.36 cm^−1^, respectively. The presence of epoxy and carboxyl groups on the surface of GO implies that the original conjugated pi-orbital system in graphite was damaged, resulting in the formation of additional reactive sites^36^. Moreover, the FT-IR of Gel@GO-F-ZrSiO_4_@Gel nanobiosorbent reveals a number of related distinct functional groups and peaks to gelatin at 1636, 1548, and 1407 cm^−1^ which directly correlated to the various forms of amide bonds in gelatin, with three other unique peaks at 429, 602, and 861 cm^−1^ corresponding to metal–oxygen bonding of either Si–O or Zr–O in ZrSiO_4_^43^. Therefore, the presence of these groups of functionalities confirms the successful preparation of the designed Gel@GO-F-ZrSiO_4_@Gel nanobiosorbent.

**Figure 3:**
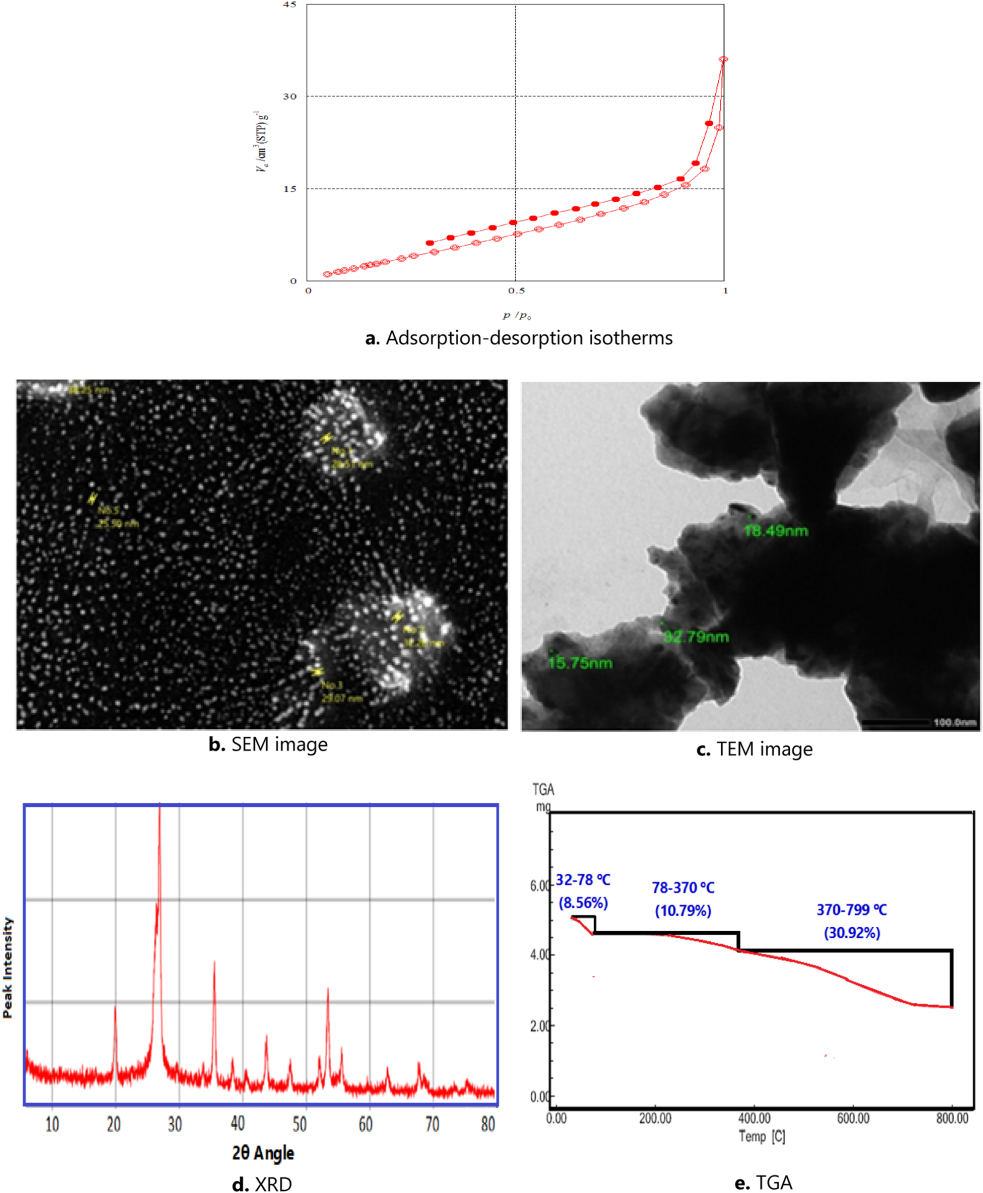
Different characterization patterns of Gel@GO-F-ZrSiO_4_@Gel.

N_2_-adsorption–desorption analysis via BET and Barrett-Joyner-Halenda (BJH) methods was used to figure out the surface area and pore size distribution in the Gel@GO-F-ZrSiO_4_@Gel nanobiosorbent. Figure 3a depicts the N_2_ adsorption–desorption isotherms curve. The Gel@GO-F-ZrSiO_4_@Gel nanobiosorbent exhibited a BET surface area of 219.46 m^2^/g, while the cumulative pore data by Barrett-Joyner-Halenda (BJH) adsorption and the average pore size were found to correspond to 4.6916 × 10^–2^ cm^3^/g and 1.22 nm, respectively.

The assembled Gel@GO-F-ZrSiO_4_@Gel nanobiosorbent was then analyzed by both SEM and HR-TEM to characterize the surface morphology as well as the shape and particles of Gel@GO-F-ZrSiO_4_@Gel nanobiosorbent and these two approaches validated the significant transformation as shown in Fig. 3b. The SEM image revealed spherical particles based on the SEM image with an average size range 15.75–32.79 nm based on the HR-TEM image as represented in Fig. 3c.

The acquired XRD pattern Gel@GO-F-ZrSiO_4_@Gel nanobiosorbent is represented in Fig. 3d and this is characterized by the existence of number of significate sharp peaks at 2θ angle = 20.1°, 26.3° 26.61°, 35.46°, 43.5°, 47°, 52°, 54°, 56°, 63° and 68°. The peak at 2θ = 26.3° is directly referring to the GO^44^, while the existence of prominent crests at 2θ = 26.61° and 35.46° are referring to the XRD peaks in ZrSiO_4_^45^. The diffraction peak at 2θ angle = 20.1°, in the XRD pattern is referring to the presence of gelatin structure in the assembled Gel@GO-F-ZrSiO_4_@Gel nanobiosorbent. Therefore, the collected data from the XRD pattern give a valuable evidence for the successful preparation of the aimed nanobiosorbent.

The TGA technique was utilized to investigate the thermal stability/degradation processes of Gel@GO-F-ZrSiO_4_@Gel nanobiosorbent. As demonstrated in Fig. 3e, the TGA-thermogram of Nano Gel@GO-F-ZrSiO_4_@Gel refers to the existence of three distinct thermal degradation stages. With a percentage loss of 8.56%, the first step (32–78 °C) could be related to water molecules desorption from the nanobiosorbent surface. The percentage loss values for the second and third degradation stages at 78–370 °C and 370–799 °C were determined to correspond to 10.79%and 30.92%, respectively which are mostly concerned with the heat breakdown of organic molecules from the loaded GO and gelatin. Finally, the total percentage decomposition from these three degradation steps are comprising a 50.27%.

### Batch removal studies of BF dye

Batch removal study of BF dye as the selected adsorbate onto Gel@GO-F-ZrSiO_4_@Gel nanobiosorbent as the selected adsorbent is generally controlled and influenced by the acting experimental conditions that favor binding of these two adsorption species. Therefore, these factors as contact reaction pH, time and temperature, initial adsorbate concentration, adsorbent mass, interfering ions must be fully investigated, monitored and optimized. The collected results from these factors can be also used to figure out the most suitable and valid mechanism for binding of BF dye onto Gel@GO-F-ZrSiO_4_@Gel nanobiosorbent based on the evaluation of kinetics models, adsorption isotherms and thermodynamic parameter^22^. The following sections will describe in details the contribution of these outlined experimental parameters.

#### Biosorptive removal of BF dye under the impact of various pHs

One of the most important factors in evaluation of the removal process of organic dyes from aqueous solutions by adsorption technique is mainly related to the pH of contact solution. This factor measures the efficiency and effectiveness of Gel@GO-F-ZrSiO_4_@Gel nanobiosorbent in binding with BF dye under various conditions of protonation and deprotonation^36^. Generally, the medium pH affects the species of the target BF dye and the degree of protonation of the loaded functional groups on the surface of Gel@GO-F-ZrSiO_4_@Gel nanobiosorbent. Therefore, a wide reaction pH range was monitored and optimized between pH 2–10 to estimate the removal progress of BF dye by the investigated Gel@GO-F-ZrSiO_4_@Gel nanobiosorbent and results of this study are compiled in Table 2 for two different initial concentrations of BF dye (5 mg L^−1^ and 10 mg L^−1^). It is evident that the biosorptive removal values of BF dye (%) onto Gel@GO-F-ZrSiO_4_@Gel were found very low at pH 2.0 condition to produce 36.0 and 24.2% using 5 mg L^−1^ and 10 mg L^−1^, respectively. This behavior could be mainly correlated to the possible repulsive forces between positively charged BF dye molecules and protonated surface of Gel@GO-F-ZrSiO_4_@Gel nanobiosorbent^25^. However, by the gradual increase in the pH value of contract solution from pH 4.0 to pH 6.0, The percentage biosorptive removal values were found to gradually increase to reach near optimum at 92.8–94.0% to confirm that the surface of Gel@GO-F-ZrSiO_4_@Gel nanobiosorbent started to loss its positively charges or protonation. It is also evident that the maximum biosorptive removal values of BF dye using 5 mg L^−1^ and 10 mg L^−1^ were established as 96.0 and 95.2%, respectively to confirm that the optimum pH condition from biosorption of BF dye from aqueous solution is the recommended neutral pH 7 condition. Finally, at higher pH conditions (pH 8.0 and 9.0), the biosorptive removal efficiency of BF dye by Gel@GO-F-ZrSiO_4_@Gel nanobiosorbent was found high by providing 89.8–91.5% and 84.0–94.5% for 5 mg L^−1^ and 10 mg L^−1^, respectively. To confirm the removal performance and efficiency of Gel@GO-F-ZrSiO_4_@Gel, the two constituent units in the assembled nanobiosorbent viz., Gel@GO and Gel@ZrSiO_4_ were tested for BF dye removal from aqueous solution under the same conditions and the results referred to 91.7% and 88.9%, as the maximum removal values, respectively.

**Table 2:** Biosorptive removal of BF dye by Gel@GO-F-ZrSiO_4_@Gel at different pHs.

BF dye concentration	Biosorptive removal of BF dye (%) at different pH conditions*								
	pH 2	pH 3	pH 4	pH 5	pH 6	pH 7	pH 8	pH 9	pH 10
5 mg L^-1^	36.0	87.2	93.8	94.0	95.4	96.0	91.5	89.8	87.6
10 mg L^-1^	24.2	73.2	89.2	92.8	94.0	95.2	94.5	94.0	93.9

*Values are based on triplicate analysis with ± 0.2–0.6%.

#### Biosorptive removal of BF dye under the impact of various masses

**Figure 4:**
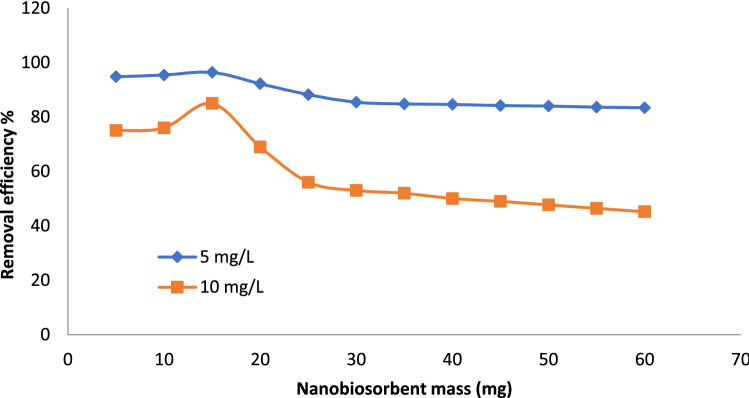
Influence of Gel@GO-F-ZrSiO_4_@Gel mass on biosorptive removal of BF dye. Removal efficiency values (%) are based on triplicate analysis with ± 0.3–0.8% (mass = 5–60 mg, dye volume = 20 mL, dye concentration = 5 and 10 mg L^-1^, shaking time = 30 min and centrifugation time = 20 min).

Biosorption of various pollutants either organic as BF dye or inorganic was found to be heavily dependent the biosorbent dosage as an important parameter because it influences the removal capacity for a given pollutant^46^. Therefore, the mass factor of Gel@GO-F-ZrSiO_4_@Gel nanobiosorbent was monitored by covering a wide range (5–60 mg) and using two different initial BF dye concentrations as 5 mg L^−1^ and 10 mg L^−1^ with the selected optimum pH 7.0. The results of this study are illustrated in Fig. 4 and clearly refer that the change in mass of Gel@GO-F-ZrSiO_4_@Gel nanobiosorbent was found to show a direct impact on the biosorptive removal (%) of BF dye. Initially, the removal values of BF dye (5 mg L^−1^ and 10 mg L^−1^) were found to increase upon increasing the mass of Gel@GO-F-ZrSiO_4_@Gel from 5 to 15 mg. It was concluded that at using 10 mg mass of Gel@GO-F-ZrSiO_4_@Gel nanobiosorbent, the maximal biosorptive removal of BF dye were identified as 98.4 and 89.2% by using 5 and 10 mg L^−1^ concentrations, respectively. Therefore, the characterized 10 mg mass of Gel@GO-F-ZrSiO_4_@Gel biosorbent is listed as the optimum mass condition for removal of BF dye from aqueous solution. This trend is mainly correlated to the high availability of nanobiosorbent interacting surface active sites for binding with BF dye molecules^47^. Finally, gradual decrease behaviors of the biosorptive removal efficiency of BF dye (%) by Gel@GO-F-ZrSiO_4_@Gel nanobiosorbent were evident by increasing the nanobiosorbent mass more than 20 mg and this trend is mainly related to the possible decrease in surface area and exposed surface active sites via aggregation of Gel@GO-F-ZrSiO_4_@Gel particles^41^.

#### Biosorptive removal of BF dye under various time impact conditions and kinetic evaluation

**Figure 5:**
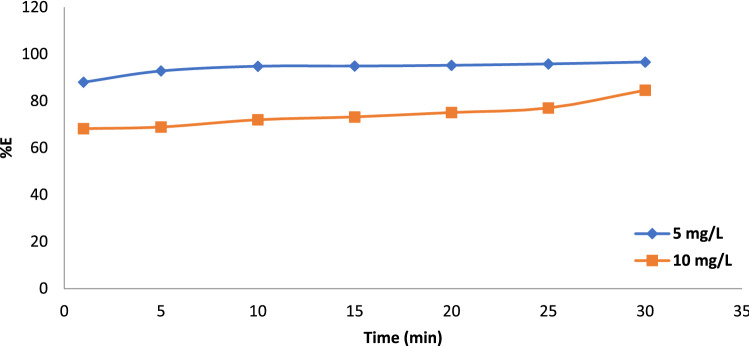
Influence of contact time on biosorptive removal of BF dye by Gel@GO-F-ZrSiO_4_@Gel. Removal efficiency values (%) are based on triplicate analysis with ± 0.1–0.5% (shaking time = 1–30 min. dye volume = 20 mL, dye concentration = 5 and 10 mg L^-1^, mass = 15 mg and centrifugation time = 20 min).

The time impact condition on the effectiveness of BF dye removal by Gel@GO-F-ZrSiO_4_@Gel nanobiosorbent was investigated in a time range from 1 to 30 min as demonstrated in Fig. 5 and the collected results refer to effectiveness and improvement in biosorptive removal of BF dye as the contact reaction time increase. Therefore, the investigated 5 and 10 mg L^−1^ BF dye concentrations were characterized to provide removal percentages corresponding to 88.0% and 68.2%, respectively after only 1 min to confirm the rapidness of binding process between BF dye and Gel@GO-F-ZrSiO_4_@Gel nanobiosorbent and these were increased to maximum values of 96.6% and 84.6% at equilibrium time 30 min, respectively.

**Figure 6:**
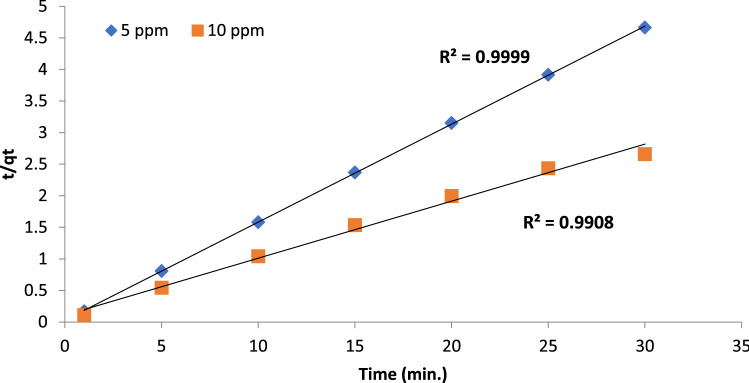
Pseudo-second order model of BF by Gel@GO-F-ZrSiO_4_@Gel.

Various kinetic models were investigated to study the kinetic behavior and parameters of possible interaction process between BF dye and Gel@GO-F-ZrSiO_4_@Gel nanobiosorbent to characterize the most valuable and valid model to describe the removal of BF dye and it is well known that the computed R^2^ value influences the kinetic parameters. In general, a kinetic model with the maximum R^2^ may be utilized to represent the biosorptive removal processes of BF dye onto Gel@GO-F-ZrSiO_4_@Gel nanobiosorbent. The *pseudo*-first order kinetic model^48^ is the first to be analyzed, and its related equation is listed and defined in Table 3. The slope k_1_ value was determined with correlation coefficient R^2^ values for 5 and 10 mg L^−1^ BF dye concentrations were corresponded to 0.893 and 0.982, respectively as given in Table 4. Therefore, one can conclude that the *pseudo* first order was not efficiently assigned to well describe the biosorptive removal process of BF dye. In addition, more confirmation of this was also related to identified removal capacity at equilibrium (q_ecal_), which is not matched with the determined experimental values (q_exp_). On the other hand, the adsorption kinetics of BF using 5 and 10 mg L^−1^ onto Gel@GO-F-ZrSiO_4_@Gel nanobiosorbent was found to strongly follow the pseudo-second-order model with R^2^ = 0.999 and 0.990, respectively as represented in Fig. 6 and Table 4. Additionally, the mechanism by intraparticle diffusion model for biosorptive removal of BF dye is thought to follow two major steps. The first stage relies on the transferred dye molecules from the aqueous solution to the surface of Gel@GO-F-ZrSiO_4_@Gel nanobiosorbent, while the second step is based on the distributed dye molecules into the Gel@GO-F-ZrSiO_4_@Gel nanobiosorbent pores^49^. The amount of removed dye by this model is proportional to t^1/2^ rather than contact duration. The application of this model on removal of BF dye onto Gel@GO-F-ZrSiO_4_@Gel nanobiosorbent using 5 and 10 mg L^−1^ referred to correlation coefficients 0.805 and 0.849, respectively to confirm its invalidity to describe this type of reaction. Finally, the Elovich model was identified to provide R^2^ = 0.632 and R^2^ = 0.868 by using 5 and 10 mg L^−1^ BF concentrations, respectively^41^. The collected and outlined results in Table 4 shows the magnitudes of correlation coefficients for the four investigated kinetic models and thus can be used to confirm that the biosorptive removal of BF dye onto Gel@GO-F-ZrSiO_4_@Gel nanobiosorbent was matches perfectly was well matched with the pseudo-second order model providing a chemisorption process^36^.

**Table 3:** Evaluated kinetic models for biosorptive removal of BF dye by Gel@GO-F-ZrSiO_4_@Gel.

Kinetic model	Linear equation	Parameter definition	Plot
Pseudo-first order	ln (qe − qt) = ln qe − k_1_t	qe and qt are the eliminated quantity of BF dye (mg g^−1^) at equilibrium and time t (min, respectively, while k_1_ is pseudo-first order rate constant (min^−1^)	ln(qe − qt) versus time (t)
Pseudo-second order	t/qt = 1/k_2_qe^2^ + t/qe	qe and qt are the eliminated quantity of BF (mg g^−1^) at equilibrium and at time t (min), respectively, while k_2_ is the pseudo second order rate constant (g/(mg min)	(t/qt) versus time (t)
Intraparticle diffusion	qt = k_id_ t ^½^ + C	kid is the intraparticle diffusion rate constant (mg g^−1^ min ^−1/2^). C is the boundary layer thickness (mg g^−1^)	(q_t_) against (t^1/2^)
Elovich	q_t_ =$\frac{1}{\upbeta }$ ln(αβ) + $\frac{1}{\upbeta }$ ln t	β is the activation energy, α is the initial elimination rate (mg g^−1^ min) and the surface coverage of chemisorption	(q_t_) versus ln t

**Table 4:** Computed kinetic parameters for biosorptive removal of BF dye by Gel@GO-F-ZrSiO_4_@Gel.

Kinetic model	BF dye (mg/L)	
	5.0 mg L^-1^	10.0 mg L^-1^
Pseudo-first order		
q_e_ (mg g^−1^)(exp.)	2.273	2.353
q_e_ (mg g^−1^)(calc.)	6.434	11.274
k_1_ (min.^−1^)	0.09	0.032
R^2^	0.893	0.982
Pseudo-second order		
q_e_ (mg g^−1^)(exp.)	6.4516	11.111
q_e_ (mg g^−1^)(calc.)	6.4337	11.274
k_2_ (min.^−1^)	0.8008	0.076
R^2^	0.999	0.990
Intraparticle diffusion		
K_id_(mg.g^−1^ min^-1/2^)	0.419	0.113
C	8.372	5.854
R^2^	0.805	0.849
Elovich		
α (mg g^−1^ min^−1^)	21868999.63	9.877*10^14^
β (mg g^−1^)	2.016	6.173
R^2^	0.632	0.868

#### Biosorptive removal of BF dye under various initial dye concentration and kinetic evaluation of the adsorption isotherms

**Figure 7:**
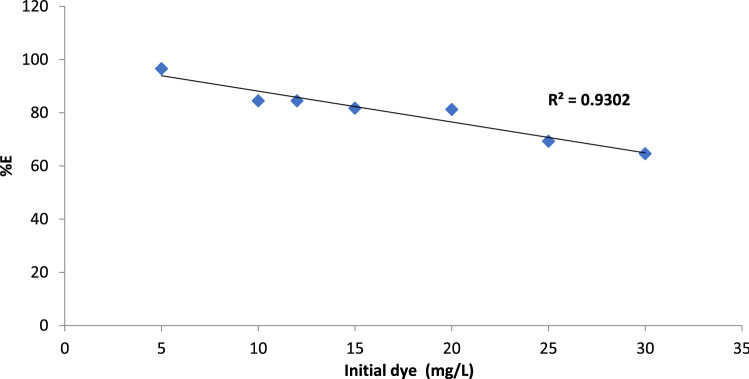
Influence of initial concentration on biosorptive removal of BF dye by Gel@GO-F-ZrSiO_4_@Gel.

**Figure 8:**
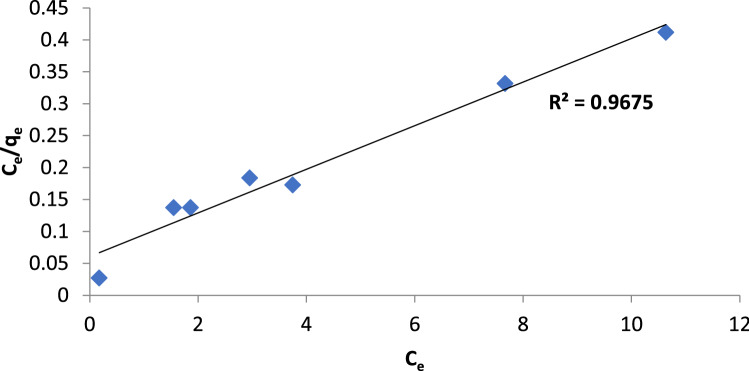
Langmuir isotherm of BF dye by Gel@GO-F-ZrSiO_4_@Gel.

It is well known that a certain adsorbent amount can bind with a specific amount of BF dye, therefore it is important to outline that the initial concentration of dye pollutant in aqueous solution is expect to play a crucial role in the adsorption process^50^. Also, it is well-documented that the adsorption reaction is controlled by the aggregation effect and the competition between the Bf dye molecules on the active sites of Gel@GO-F-ZrSiO_4_@Gel nanobiosorbent. The impact of BF dye initial concentration on the effectiveness of Gel@GO-F-ZrSiO_4_@Gel nanobiosorbent was studied and monitored by selection a concentration range between 5 to 30 mg L^−1^, and the outcomes of this study are shown in Fig. 7. The proportion of dye removal was decreased as the dye concentration increased to confirm the possible decrease in the number of reactive sites that are available on the surface of Gel@GO-F-ZrSiO_4_@Gel nanobiosorbent to lead to a direct drop in the binding efficiency between the two interacting species. Therefore, the maximum percentage removal of BF dye (%R = 98.2%) was established upon using 5 mg L^−1^ as the initial dye concentration, whilst the minimum BF dye removal (%R = 72.3%) was established upon using 30 mg L^−1^ as illustrated in Fig. 8.

To examine the best fitting of the experimental results and characterize the adsorption equilibrium, four different adsorption isotherm models were testified and evaluated in this study as Langmuir, Freundlich, and Dubinin–Radushkevich (D–R) models^51^. Irving Langmuir investigated the Langmuir theory on the basis of a monolayer biosorption process and on a homogeneous surface with no integration between the molecules that have been eliminated^52^. This model is governed by the listed equation in Table 5 as a guide and a straight line was detected for removal of BF dye onto Gel@GO-F-ZrSiO_4_@Gel nanobiosorbent providing an excellent correlation value R^2^ 0.9675 (Fig. 8) to denote that the Langmuir theory may be applied to best describe the removal of BF dye by the assembled Gel@GO-F-ZrSiO_4_@Gel nanobiosorbent. The slope and intercept were used to calculate the q_max_ and b values, as outlined in Table 6. The equation for the separation factor (RL, Table 5), which is utilized to figure out what kind of biosorption technique is to favor. If 0 < R_L_ < 1, the approach is favorable; if R_L_ = 0, the approach is irreversible; if R_L_ > 1, the approach is unfavorable and if R_L_ = 1, it means that the process is linear, as shown in Table 6. The identified R_L_ values in this work for removal of BF dye onto Gel@GO-F-ZrSiO_4_@Gel nanobiosorbent were 0.102–0.0185, indicating a good biosorption procedure. On the other hand, Freundlich adsorption isotherm was set up by an empirical formula to predict a multilayer biosorption of the dye molecules on a heterogeneous surface with uneven available sites and various biosorption energies^43^ as listed in Table 5. Based on the computed correlation coefficient R^2^ was found to correspond to 0.955 according to this model to confirm that Freundlich adsorption isotherm model is less valid to apply for removal BF dye onto Gel@GO-F-ZrSiO_4_@Gel nanobiosorbent when compared to Langmuir theory.

**Table 5:** Different evaluated isotherm models for biosorptive removal of BF dye by Gel@GO-F-ZrSiO_4_@Gel.

Adsorption isotherm	Models	Equation linear form Parameter definition	Plot
Langmuir	C_e_/q_e_ = (1/q_max_) k_1_ + C_e_/q_max_ b R_L_ = 1/1 + b C_o_	C_o_ and C_e_ is for to the initial and equilibrium concentrations (mg/L), respectively. q_e_ is the amount of eliminated dye at equilibrium(mg g^−1^). q_max_ (mg g^−1^) the maximum elimination efficiency which is used to investigation elimination energy and elimination capacity and b (L mg^−1^) is Langmuir constants	Ce/qe versus Ce
Freundlich	lnq_e_ = ln k_f_ + 1/n ln C_e_	q_e_ is the amount of eliminated dye which is connected to the equilibrium concentration of dye in solution and C_e_ is the equilibrium dye concentration. K_F_ (mg g^−1^) is Freundlich constant, n is the intensity of the adsorbent	log qe versus log Ce
Temkin	q_e_ = (RT/b_T_) ln a_T_ + (RT/b_T_) ln C_e_ q_e_ = B ln a_T_ + B ln C_e_ B = RT/b_T_	b_T_ (mg/L) is the Temkin isotherm constant, a_T_ (L/g) is the Temkin isotherm equilibrium binding constant and B is constant represents the heat of elimination (J/mol)	q_e_ versus ln C_e_
Dubinin–Radushkevich (D–R)	ln qe = lnqs − (Kad Ɛ^2^) ε = RT ln (1 + 1/Ce) Es = $\frac{1}{\sqrt{2Kad}}$	K_ad_ the D–R isotherm constant which is related to the elimination free energy per mole of the dye (mol^2^/kJ^2^). q_s_ (mg g^−1^) is the theoretical fullness efficiency ε is the Polanyi potential which is based on equilibrium, R is the universal gas constant (8.314 J/mol K^−1^) and T absolute temp. Kelvin	ln qe versus ε^2^

**Table 6:** Computed isotherm parameters for biosorptive removal of BF dye by Gel@GO-F-ZrSiO_4_@Gel.

Isotherm model	Isotherm parameters	BF
Langmuir	q_max_ (mg g^−1^)	29.4
	b (L mg^−1^)	1.765
	R_L_	0.102–0.0185
	R^2^	0.967
Freundlich	n	2.84
	K_f_ (L. mg^−1^)	2.86
	R^2^	0.955
Temkin	a_T_ (L. g^−1^)	13.31
	b_T_ (J/mol)	509.4
	B	4.864
	R^2^	0.879
Dubinin-Radushkevich	q_s_ (mg g^−1^)	18.45
	K_ad_ (mol^2^/j^2^)	5*10^–8^
	E_s_ (kJ mol^−1^)	3162.3
	R^2^	0.682

According to the Temkin isotherm model^53^, the binding energies are arranged in a uniform distribution. By excluding the very high and very low initial dye concentration, this model suggests a linear decrease (not a logarithmic one) in the adsorption heat as the Gel@GO-F-ZrSiO_4_@Gel nanobiosorbent is increasingly covered by the BF dye, due to direct interactions between the two species. The Temkin model also gives an indication of whether adsorption is chemically or physically controlled. According to the Temkin isotherm (Table 5), the dye removal was found to decrease linearly with the increase in surface coverage of Gel@GO-F-ZrSiO_4_@Gel nanobiosorbent and the computed correlation coefficient R^2^ was found to correspond to 0.879 refer that this model is invalid to account for the biosorptive removal BF dye onto Gel@GO-F-ZrSiO_4_@Gel nanobiosorbent.

Finally, the Dubinin–Radushkevich (D-R) isotherm (Table 5) was also employed to estimate the BF dye biosorption process onto Gel@GO-F-ZrSiO_4_@Gel nanobiosorbent, porosity, and the biosorption energy (Es)^54^. This model is more general in nature, especially when compared to the Langmuir theory as it is not relating the adsorption process to a homogenous layer formation on the surface of Gel@GO-F-ZrSiO_4_@Gel nanobiosorbent. Another dimension of D-R does is related to independent steric hindrance between the two interacting materials. The D-R isotherm equation can determine the mean free energy of adsorption, expressed as Es and measured in kJ mol^−1^ to help in understanding the type of the adsorption mechanism. The identified correlation coefficient value (R^2^) by this model was 0.682 and the computed Es were 3162.3 kJ mol^−1^ (Table 6). It was reported that when the magnitude of Es is larger than 16 (kJmol^−1^), the reaction is considered chemisorption^4^ and therefore, one can conclude that the biosorptive removal of BF dye onto Gel@GO-F-ZrSiO_4_@Gel nanobiosorbent was based on a chemisorption process.

Finally, the computed findings and results in Table 6 refer that the best model to describe biosorptive removal of BF dye onto Gel@GO-F-ZrSiO_4_@Gel nanobiosorbent is based on the Langmuir adsorption isotherm model.

#### Interfering cations on biosorptive removal of BF dye onto Gel@GO-F-ZrSiO_4_@Gel

**Figure 9:**
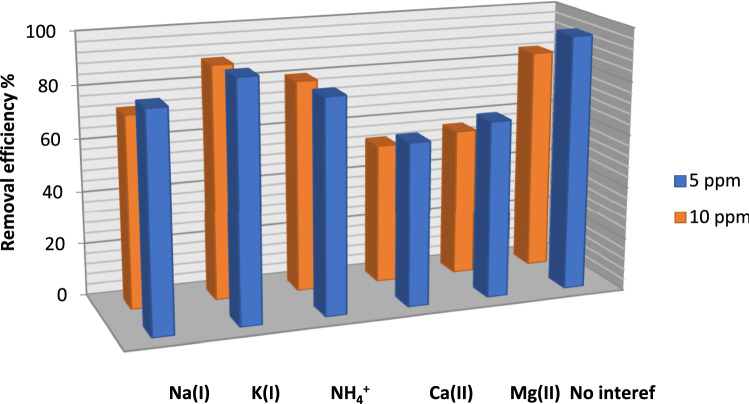
Influence of interfering species on biosorptive removal of BF dye by Gel@GO-F-ZrSiO_4_@Gel. Removal efficiency values (%) are based on triplicate analysis with ± 0.8–1.4%.

BF dye possesses a cationic structure with reactive positively charged functional groups. In general, a cationic species in solution can easily interfere in the biosorptive removal of BF as a cationic dye onto Gel@GO-F-ZrSiO_4_@Gel and therefore, the entire biosorption process of BF dyes may be disturbed. Different natural water resources are generally containing various types of cationic and anionic species. Accordingly, the effect of cationic species as Na^+^, K^+^, Mg^2+^, Ca^2+^, NH_4_^+^ on the biosorptive removal of BF dye onto Gel@GO-F-ZrSiO_4_@Gel was investigated in this section and the results are illustrated in Fig. 9. Comparison of the removal efficiency (R%), the experimental results showed that the selected co-existing cationic species was found to influence the removal of BF dyes with various degree of contributions. In presence of metal cations, the adsorption sites on the surface of Gel@GO-F-ZrSiO_4_@Gel nanobiosorbent were minimized and thus decreasing its efficient bonding with BF dye. Moreover, the ionic radii and ionic charge of the metal ions along with the type of functional groups involved on the adsorbent surface may be the reasons for such change in the adsorption efficiency of Gel@GO-F-ZrSiO_4_@Gel. This means that the higher the number of positive charges, the higher the number of attracted negatively charged functional groups^55^. Based on this investigation, the interfering cationic species were arranged in order of decreasing from the least affecting ion to the most affecting cation as shown in Fig. 9, K(I) exhibited the least impact on the biosorptive removal of BF dye onto Gel@GO-F-ZrSiO_4_@Gel providing 90.4% and 88.6% for BF dye for 5 and 10 mg L^−1^ concentrations, respectively. However, Ca(II) and Mg(II) cations were characterized to exhibit the most significant interfering impact on the percentages of BF dye biosorptive removal by Gel@GO-F-ZrSiO_4_@Gel providing 62.0 and 64.5 using 10 mg L^−1^ as well as 53.4% and 55.1% using 5 mg L^−1^ dye concentrations, respectively.

#### The impact of reaction temperature on efficacy of biosorption of BF dye

**Figure 10:**
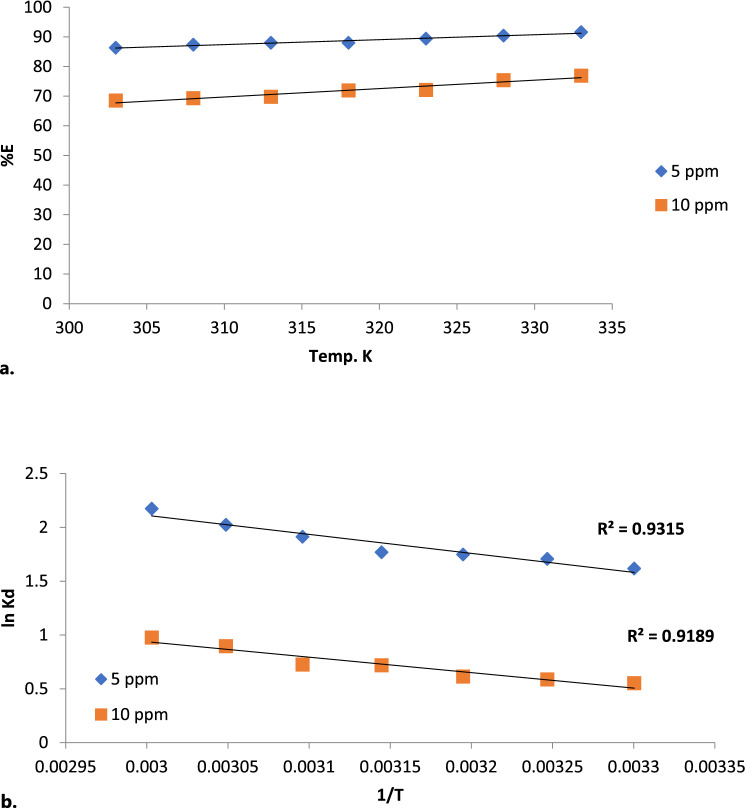
(**a**) Influence of temperature on biosorptive removal of BF dye by Gel@GO-F-ZrSiO_4_@Gel. Removal efficiency values (%) are based on triplicate analysis with ± 0.3–1.0%. (**b**) Vant Hoff plot for biosorptive removal of BF dye by Gel@GO-F-ZrSiO_4_@Gel.

The nature of biosorption process, whether endothermic or exothermic, can be investigated and confirmed from the change in reaction temperature and its variation effect on the BF dye biosorptive removal efficacy by Gel@GO-F-ZrSiO_4_@Gel. Significantly, Fig. 10a depicts reaction temperature impact and shows an increase in the removal percentage with increasing the reaction temperature. The percentage of BF dye biosorptive removal efficacy was found to gradually increase as the temperature rises from 303 to 333 K. This indicates that the BF dye biosorptive removal process onto Gel@GO-F-ZrSiO_4_@Gel nanobiosorbent is an endothermic in nature. The adsorption and biosorption behaviors are commonly understood by computing a number of thermodynamic parameters utilizing the Vant Hoff plot (Fig. 10b) for the BF dye biosorptive removal efficacy by Gel@GO-F-ZrSiO_4_@Gel and these include (ΔS°), (ΔH°) and (ΔG°) as represented by Eqs. (2–5)^56^.

2 $$\Delta G^{o} = - RT\ln K_{d}$$

3 $$\Delta G^{o} = \Delta H^{o} - T\Delta S^{o}$$

4 $$\ln K_{d} = \frac{{\Delta S^{o} }}{R} - \frac{{\Delta H^{o} }}{RT}$$

5 $$K_{d} = \frac{{q_{e} }}{{C_{e} }}$$

K_D_ (L/g) represents the biosorption equilibrium constant, R denotes the general gas constant (J/mol.k), T denotes temperature (K), C_e_ is the BF dye concentration (mg L^−1^) at equilibrium, and q_e_ is the amount of BF dye removed (mg g^−1^). The collected data in Table 7 refer to a spontaneous biosorption reaction as established by the negative magnitude of (ΔG°). The negative values of ΔG^o^ for all tested temperatures suggest that the adsorption of BF dye onto Gel@GO-F-ZrSiO_4_@Gel is based on a feasible and spontaneous reaction Moreover, it is evident higher reaction temperatures were found to enhance the biosorption of BF dye onto Gel@GO-F-ZrSiO_4_@Gel nanobiosorbent. Furthermore, the positive value of (ΔS°) suggested an increase in unpredictability by the BF dye biosorptive removal process onto Gel@GO-F-ZrSiO_4_@Gel nanobiosorbent, and the positive magnitude of (ΔH°) corroborated that the biosorption reaction is endothermic.

**Table 7:** Thermodynamic parameters for biosorptive removal of BF dye by Gel@GO-F-ZrSiO_4_@Gel.

BF dye (mg L^-1^)	Temp (K)	Thermodynamic parameters		
		Δ G° (KJmol^− 1^)	Δ H° (kJmol^− 1^)	ΔS° (Jmol^− 1^ K^− 1^)
5 mg L^− 1^	303	− 4.85	31.05	83.65
	308	− 4.40		
	313	− 4.02		
	318	− 3.72		
	323	− 3.51		
	318	− 3.39		
	333	− 2.81		
10 mg L^− 1^	303	− 3.43	− 22.02	− 59.31
	308	− 3.05		
	313	− 2.72		
	318	− 2.38		
	323	− 2.37		
	328	− 2.10		
	333	− 1.65		

#### Biosorptive removal of BF dye from water samples

The practical application of the investigated Gel@GO-F-ZrSiO_4_@Gel in BF dye biosorptive removal from real water samples was explored in this study as the final. In this test, the batch equilibrium technique was used to make sure that the prepared bionanocomposite is capable of removing the investigated BF dye pollutant from different water sources (tap water, sea water and industrial wastewater). The optimum experimental conditions were applied for water samples that spiked with 5 and 10 mg L^−1^ BF dye and Table 8 lists out the collected data from this study. It is evident that the BF dye biosorptive removal efficacy by Gel@GO-F-ZrSiO_4_@Gel from spiked 5 mg L^−1^ BF dye in tap water, sea water, and industrial wastewater were 92.3%, 87.4%, and 92.8%, respectively. Similarly, spiking 10 mg L^−1^ BF dye in tap water, sea water, and industrial wastewater were found to yield 80.3%, 74.1%, and 81.4% removal by the action of Gel@GO-F-ZrSiO_4_@Gel, respectively. As a result, the findings of this study reveal that the assembled and evaluated Gel@GO-F-ZrSiO_4_@Gel can be categorized as a valid and applicable nanobiosorbent for the instantaneous removal of cationic BF dye pollutant with high biosorption behavior from water.

**Table 8:** Application of Gel@GO-F-ZrSiO_4_@Gel in biosorptive removal of BF dye from real water samples.

Water sample	Biosorptive removal of BF dye (%)*	
	5.0 mg L^-1^	10.0 mg L^-1^
Tap water	92.3	80.3
Seawater	87.4	74.1
Wastewater	92.8	81.4

*Values are based on triplicate analysis with ± 0.2–0.6%.

## Conclusion

A sustainable and innovative nanobiosorbent was assembled from gelatin, graphene oxide and zirconium silicate via formaldehyde crosslinking for the formation of Gel@GO-F-ZrSiO_4_@Gel. The produced nanobiosorbent was characterized by number of techniques as FT-IR which confirmed several incorporated surface reactive functionalities as -OH, = NH, –NH_2_, -COOH and C = O. SEM and TEM analyses of Gel@GO-F-ZrSiO_4_@Gel nanobiosorbent confirmed the particle size 15.75- 32.79 nm and the BET surface area corresponded to 219.46 m^2^ g^-1^. The as-prepared Gel@GO-F-ZrSiO_4_@Gel was aimed to explore its biosorptive removal of basic fuchsin pollutant as an example of a widely applicable dye via monitoring various parameters and the optimized conditions were established as under the influence of pH (6–7), reaction time (30), nanobiosorbent dosage (10 mg), temperature (60 °C). The Thermodynamic parameters demonstrated spontaneous and endothermic adsorption reaction between BF dye and Gel@GO-F-ZrSiO_4_@Gel to take place. Chemisorption was listed as the predominant adsorption mechanism with multilayers formation in accordance with the hypotheses of pseudo-second order and Freundlich models. The investigated Gel@GO-F-ZrSiO_4_@Gel nanobiosorbent was also successfully tested to affirm its applicability of in biosorptive removal of BF pollutant from real waters by the batch biosorptive technique. The evaluated Gel@GO-F-ZrSiO_4_@Gel nanobiosorbent was compared versus other previously reported adsorbents for removal of BF dye and found more efficient and superior with respect to the optimum mass (15 mg) and optimum equilibration time (10 min) as listed in Table 9^57–62^. Finally, the outcomes and findings of this study clearly indicate that optimized sustainable and innovative Gel@GO-F-ZrSiO_4_@Gel biosorbent could lead to significant influences on remediation of industrial effluents containing BF pollutant with superior efficiency characters.

**Table 9:** Comparison of other adsorbents versus Gel@GO-F-ZrSiO_4_@Gel for removal efficiency of Basic fuchsine dye.

Adsorbent	Removal conditions	Adsorption capacity	Refs.
Defatted carica papaya seeds	Optimum mass = 6 g/L Optimum pH = 3 , Optimum Time = 40 min	95.08%	^57^
Mussel shell biomass waste	Dye concentration = 50–200 mg L^−1^, Adsorbent dose = 500 mg, Contact time = 240 min, Optimum pH = 6	Removal = 91.2%	^58^
HCl treated malted sorghum mash	Optimum time = 30 min, Optimum pH = 6–8 Adsorbent dose = 50–400 mg	Removal capacity = 58.48 mg/g	^59^
Graphite oxide modified aromatic polyurethane foam	Time = 10 h, pH = 8, Adsorbent dose = 50 mg mL^-1^	Removal = 84.6–99%	^60^
Polyacrylamide/laponite nanocomposite hydrogels	pH = 7.5, Adsorbent dose = 2.5 g	Removal capacity = 22.60 mg/g	^61^
Recyclable Fe3O4@CD magnetic microspheres	Optimum dye concentration = 25 mg/L optimum adsorbent dosage = 100 mg Optimum pH = 7	Removal = 95%	^62^
TiO_2_/MWCNTs	Optimum time = 40 min Optimum pH = 6	Removal = 98.0%	^25^
Gelatin@graphene oxide-crosslinked-zirconium silicate@gelatin	Dye concentration = 5–10 mg L^-1^ Optimum pH = 7 Optimum mass = 15 mg Optimum time = 10 min	Removal = 95.2–96.0%	This work

## Supplementary Information

Supplementary Information.

## Data Availability

All data will be available and request for materials should be addressed to Mohamed E. Mahmoud.
