# Supplementary material for: Synthesis of innovative and sustainable gelatin@graphene oxide-crosslinked-zirconium silicate@gelatin nanobiosorbent for effective biosorption of basic fuchsin dye
Source: Sci Rep. 2023 Apr 1;13:5347. doi: 10.1038/s41598-023-31584-x (PMC10067947; doi:10.1038/s41598-023-31584-x)
Supplement: Supplementary file 1 — Supplementary Information. [file 41598_2023_31584_MOESM1_ESM.docx]

**Table 1S.** Specifications of chemicals and other materials

| **Chemical name** | **Formula** | **FW (g/mol)** | **Assay** | **Company** |
| --- | --- | --- | --- | --- |
| Ultrapure graphite fine powder | C | 12 g/mol, | 99.5% 150 mesh | Lanxess, Germany |
| Gelatin | C_102_H_151_N_31_O_39_ | 2433 | >98.0 % | Elamin for Gelatine Co, Egypt |
| Potassium permanganate | KMnO_4_ | 158.03 | 99.0% | Sigma Aldrich, USA |
| Hydrogen peroxide (50%) | H_2_O_2_ | 34 | 99.0% | VWR international Ltd Poole. |
| Potassium persulfate | K_2_S_2_O_8_ | 270.31 | 98% | BH15 1 TD, England. |
| Sodium chloride | NaCl | 58.44 | 99.5% | BDH chemicals Lt. England |
| Ammonium chloride | NH_4_Cl | 53.49 | 99.8% |  |
| Potassium chloride | KCl | 74.55 | 99.5% |  |
| Hydrochloric acid | HCl | 36.46 | 99.0% |  |
| Sulfuric acid | H_2_SO_4_ | 98.08 | 98.0% |  |
| Sodium hydroxide | NaOH | 40.0 | 96.0% | Riedel de Haën |
| Ethyl alcohol | C_2_H_5_OH | 46.06 | 99.0% | Oxford, India |

**Table 2S.** Specifications of various instrumental techniques

| **Characterization** | **Instrument** | **Conditions** |
| --- | --- | --- |
| **FT-IR** | BRUKER VERTEX 70 Fourier Transform infrared spectrophotometer | in the scope 400–4500 cm^−1^ |
|  |  |  |
| **SEM** | Scanning electron microscopic JSM-6360LA, JEOL Ltd. | Using an ion sputtering coating device (JEOL-JFC-1100E) |
|  |  |  |
| **TEM** | Transmission electron microscopy,model JEOL JEM-2100F, Japan | Acquiring the images at 80 to 200 kV |
| **XRD** | The X-ray diffraction by XRD Shimadzu lab X6100, Japan | The XRD generator worked at 40 kV, 30 mA, and λ = 1 Å utilizing target Cu-Kα with secondary monochromatic.  2-Theta was started at 10° and ended at 80°.  The diffraction data was recorded with step of 0.02° and a time of 0.6 s at room temperature |
| **UV/ViS spectrophotometer** | Ultraviolet/visible spectrophotometer by V-530 JASCO | UV/ViS spectrophotometer in between the range of wavelength from 190 nm to 1100 nm was used in the absorption measurement |
| **pH-meter** | Adwa pH-meter | Standard buffers 4.01, 7.00 and 10.00 were utilized in the calibration of Adwa pH-meter which used in the measurements of solutions pH. |
